# Understanding Online and Offline Social Networks in Illness Management of Older Patients With Asthma and Chronic Obstructive Pulmonary Disease: Mixed Methods Study Using Quantitative Social Network Assessment and Qualitative Analysis

**DOI:** 10.2196/35244

**Published:** 2022-05-17

**Authors:** Andreas Andreou, Amar Dhand, Ivaylo Vassilev, Chris Griffiths, Pietro Panzarasa, Anna De Simoni

**Affiliations:** 1 Wolfson Institute of Population Health Asthma UK Centre of Applied Research Queen Mary University of London London United Kingdom; 2 Department of Neurology, Brigham & Women's Hospital, Harvard Medical School Boston, MA United States; 3 Social Networks Health and Wellbeing Research Group, School of Health Sciences, University of Southampton Southampton United Kingdom; 4 School of Business and Management, Queen Mary University of London London United Kingdom

**Keywords:** social networks, asthma, COPD, self-management, elderly, online health communities, online forums, digital health, mobile phone

## Abstract

**Background:**

Individuals’ social networks and social support are fundamental determinants of self-management and self-efficacy. In chronic respiratory conditions, social support can be promoted and optimized to facilitate the self-management of breathlessness.

**Objective:**

This study aimed to identify how online and offline social networks play a role in the health management of older patients with chronic respiratory conditions, explore the role of support from online peers in patients’ self-management, and understand the barriers to and potential benefits of digital social interventions.

**Methods:**

We recruited participants from a hospital-run singing group to a workshop in London, the United Kingdom, and adapted PERSNET, a quantitative social network assessment tool. The second workshop was replaced by telephone interviews because of the COVID-19 lockdown. The transcripts were analyzed using thematic analysis.

**Results:**

A total of 7 participants (2/7, 29%, men and 5/7, 71%, women), with an age range of 64 to 81 years, produced network maps that comprised between 5 and 10 individuals, including family members, health care professionals, colleagues, activity groups, offline and online friends, and peers. The visual maps facilitated reflections and enhanced participants’ understanding of the role of offline and online social networks in the management of chronic respiratory conditions. It also highlighted the work undertaken by the networks themselves in the self-management support. Participants with small, close-knit networks received physical, health, and emotional support, whereas those with more diverse and large networks benefited from accessing alternative and complementary sources of information. Participants in the latter type of network tended to communicate more openly and comfortably about their illness, shared the impact of their illness on their day-to-day life, and demonstrated distinct traits in terms of identity and perception of chronic disease. Participants described the potential benefits of expanding their networks to include online peers as sources of novel information, motivation, and access to supportive environments. Lack of technological skills, fear of being scammed, or preference for keeping illness-related problems for themselves and immediate family were reported by some as barriers to engaging with online peer support.

**Conclusions:**

In this small-scale study, the social network assessment tool proved feasible and acceptable. These data show the value of using a social network tool as a research tool that can help assess and understand network structure and engagement in the self-management support and could be developed into an intervention to support self-management. Patients’ preferences to share illness experiences with their online peers, as well as the contexts in which this can be acceptable, should be considered when developing and offering digital social interventions. Future studies can explore the evolution of the social networks of older people with chronic illnesses to understand whether their willingness to engage with online peers can change over time.

## Introduction

### Background

Asthma and chronic obstructive pulmonary disease (COPD) are debilitating chronic illnesses that affect not only individuals but also have a huge impact on society as a whole [1-3]. Promoting self-management and improving self-efficacy in COPD and asthma is an effective method of tackling their burden [1,2], as it can lead to better use of health care professionals’ time and enhanced self-care [2]. Similarly, a Cochrane review conducted by Gibson et al [3] showed that self-management education for adults with asthma could lead to an overall improvement in their quality of life, with a reduction in days lost from work, hospitalizations, accident and emergency department visits and unscheduled physician visits for asthma, and episodes of exacerbation of asthma at night [4].

Corbin and Strauss [5] described *self-management* as a three-step process: (1) medical management, (2) creation of new meaningful behaviors, and (3) dealing with the emotional aspects of a chronic condition. Self-management involves different types of work performed by people with long-term conditions (LTCs) and members of their networks [6]: illness related (ie, the tasks necessary to manage or treat a chronic illness and its sequelae), emotional, biographical (ie, the task of defining and maintaining an identity over the life course), and relational (ie, the tasks that are required to develop and sustain interpersonal relationships) [7]. Doing this work requires negotiating changes at the individual and network levels, which is a process shaped by individual self-efficacy, the collective efficacy of the networks of people with LTCs, and the support and resources available to individuals and within their networks [8-10]. The concept of *self-efficacy* was first described by Bandura [11] in 1977 as a person’s belief regarding whether they feel they can successfully execute particular behaviors to produce certain outcomes. Bandura [12] portrayed self-efficacy as a dynamic concept that changes over time, with expectations of personal efficacy derived from performance accomplishments, vicarious experiences, verbal persuasion, and physiological states. Patients who can withstand failures associated with acquiring the skills of a more intricate task are more likely to persevere and continue with that task [13].

As the effectiveness of self-management can be directly affected by the patient’s self-efficacy, health care providers can improve the confidence of patients in self-management by increasing their self-efficacy. A study conducted by Simpson and Jones showed that patients with COPD who are more confident in understanding and treating an exacerbation and controlling their breathlessness showed less anxiety and depression levels [14].

The fundamental determinants of self-management and self-efficacy are an individual’s social network and social support [15]. An individual’s *personal social network* comprises interpersonal connections among the individual’s family members, friends, and acquaintances and can have broad effects on health outcomes and quality of life [15]. *Social support* is defined as the relative presence or absence of psychosocial support from significant others to meet a person’s basic social needs [16] and typically comes in 4 forms: informational, emotional, instrumental, and appraisal [17]. Social support is further subdivided into two categories: *perceived social support* (ie, the belief that support is available when and if needed) and *received support* (ie, the exchange of support-related resources) [18]. Approximately 30 years ago, a causal link between social relationships and mortality was proposed [19]. Since then, numerous reviews have documented how perceived social support can influence physical health outcomes [17,20-22]. Some of the most compelling results were provided by a meta-analysis conducted by Holt-Lunstad et al [22], who concluded that perceived support was related to a significantly lower risk of all-cause mortality.

Previous literature has demonstrated that behavioral change and overall health and well-being are shaped by relationships within networks. These include weight gain [23], smoking cessation [24], happiness [25], and adherence to preventative medication [26]. One of the ways of studying the effects of social networks on behavioral and health-related properties is through the assessment of individuals’ social capital. Granovetter [27] proposed a seminal account of the structural foundations of social capital through the “strength of weak ties.” Granovetter [27] suggested that strong ties, although they foster a sense of belonging and sustain emotional support, can lead to overall fragmentation (fragmentation is defined as the proportion of mutually reachable nodes as each node is removed from the network, in other words, an inverse measure of the amount of connection redundancy in a network), whereas weaker ties amplify an individual’s access to novel information and opportunities, as they introduce the individual to nonredundant and unique knowledge pools provided by disconnected neighbors. The idea of informational nonredundancy was further reinforced by Burt [28] in his theory of structural holes (ie, the absence of direct links between individuals who share a common neighbor) and their beneficial impact on the focal node. The focal node can access complementary and nonredundant sources of information through their direct links to otherwise disconnected others [21]. In the context of LTCs, weak ties have been demonstrated to offer access to a wide range of support that deburdens strong, intimate ties and is also easy to accept and reciprocate. Compared with strong ties, weak ties in the networks of people with LTCs require less relational work to sustain them over time. Weak ties are also less threatening to an individual’s sense of self and the valued roles and responsibilities they have to others. Weak ties play a key role in navigating and negotiating relationships within networks and thus increase the collective efficacy of networks as they allow individuals to access support that is acceptable to them and to members of their networks [7,10,29].

The rapid growth of social media and web-based social platforms in recent years has allowed for new approaches to social networks and support. In addition to social platforms aimed at the general population, health-related social platforms have seen a recent upsurge in popularity [30]. Integrating social media and web-based communities in the health sector opens up new potential applications [31,32], including their use as public health surveillance tools [33-35] and health-related information sources [36,37]. A meta-analysis conducted by Laranjo et al [38] showed a positive effect of social networking site interventions on health behavior–related outcomes, encouraging future research in this area [38-40]. Moreover, there is evidence that social media interventions are effective in promoting health equity (health equity means that everyone receives individualized care to bring them to the same level of health, despite health disparities between population groups) [40-42]. Forming new social connections with individuals with similar lived experiences (such as in social media interventions) has been reported to facilitate the emergence of a sense of community and strengthen engagement with social prescriptions [43]. Taking the concept of social support a step further, Panzarasa et al [44] have proposed the concept of social-medical capital, defined as “the advantages that any user can gain from participation in the social networks provided by online health communities” (online health communities [OHCs]). In particular, these advantages include improvement in patients’ self-care and health in resource-constrained systems.

### Objectives

Web-based personal network surveys have been developed to evaluate an individual’s social network in a structured manner, translating the complexity and burdensome features of this type of questionnaire into a more usable and scalable form. Interventions aimed at modulating network composition in a social network hold the promise of a novel complementary approach to the self-management of chronic respiratory conditions. Here, we used a data collection tool that can be completed by older patients without an interviewer [45]. The objectives of this study were to adapt this previously validated social mapping network assessment tool to include online contacts to (1) understand the feasibility of using this tool to map the social networks of individuals with chronic respiratory conditions within a workshop or telephone interview; (2) identify how online and offline social networks play a role in health management for patients with chronic respiratory conditions, specifically COPD and asthma; (3) explore the role of any existing online peers in patients’ self-management; and (4) shed light on the barriers and potential benefits of digital social interventions.

## Methods

This was a mixed methods study that used quantitative social network assessment and qualitative analysis.

### Participants and Setting

Between March and July 2020, we ran a public engagement activity titled *Promoting Research in Social Media and Health* with participants attending *Singing for Breathing* sessions at the Royal London Hospital in Whitechapel, London, to inform a grant application for developing and testing a digital social intervention. Recruitment was opportunistic, targeting a group more likely to be socially engaged, as our focus was to understand online and offline social engagement in LTCs rather than socially versus not socially engaged patients. The inclusion criterion comprised adults aged >60 years with a chronic respiratory condition. Participants provided written informed consent to take part in the study and for their anonymized quotes to be reported in the publications.

A total of 2 workshops were initially planned. The first workshop took place immediately before the singing activity. Owing to the COVID-19 lockdown, the second workshop was replaced by individual telephone interviews. The workshop activity was piloted with 2 patient and public involvement (PPI) members. Through the PPI piloting, it became clear that a 1-hour workshop would not provide enough time to guide participants to fill in the web-based survey at the same time. The survey questions were instead printed on A4 sheets, presented, and read aloud to participants during the workshop so that participants could draw simultaneously. The participants drew their networks using pencils of different colors. Red markers were used to link individuals who supported participants or one another most often (strong ties), and blue markers were used for all other contacts (weak ties). Data were subsequently transferred from paper to the web-based survey of the social network assessment tool. During the COVID-19 lockdown, participants were emailed written instructions (previously piloted by the PPIs) to complete the web-based questionnaire. According to their preferences, they could fill out the web-based survey on their own time or with the researcher’s guidance by phone. They were subsequently emailed their social network maps and interviewed by phone while looking at them.

### Data Collection

We collected data by adapting PERSNET, a publicly available social network assessment tool, on a secure open-source web platform (REDCap [Research Electronic Data Capture; Vanderbilt University]) [45], which generated social network maps. We focused on individuals involved in managing their illness, whether offline or on the web (ie, social contacts not involved in the management of their respiratory condition were not collected). Maps were generated in RStudio using the data collected from the REDCap survey [46]. Participants were prompted to answer the *name generator* question (“Think about people who encourage you to stay healthy by giving you motivation, advice, or direct help. Who provides this kind of support for your health?”) thinking of whoever was involved in any aspect of the management of their respiratory conditions (eg, providing medical, practical, or emotional support during exacerbations or practical help in the long-term management of the condition, eg, with ordering and collecting medications and prompting medication taking). We asked the participants to include any important individuals: clinicians, close family members (first-degree relatives and partners), and friends (both offline and on the web). Other collected information included the description of relationships with each individual (*especially close* or *not especially close*); the relationships between each pair of people in the network (*stranger, in between* or *especially close*); those who were supporting the participant *most often*; gender, ethnicity, approximate age, and higher degree of each individual, if known; how often they communicated with each person (*daily, weekly, monthly, less often,* or *don’t know*); how many years they had known each other for (*<3 years, 3-6 years, >6 years,* or *don’t know*); the way in which the participant was connected with each individual (*spouse, family, friend, adviser, coworker,* or *other*); and how far they lived from each person (*same house, 1-5 miles, 6-15 miles, 16-50 miles*, or ≥*50 miles*).

Once the maps were created, participants had the opportunity to reflect on how their offline or online social networks played a role in managing their respiratory condition based on the visual representation of their social maps. They were also prompted to reflect on whether they were in contact with any online peers and, if not, what would the barriers and potential benefits of doing so be.

### Analysis

We performed network and qualitative analyses of the workshop and interview transcripts and triangulated the results [47].

#### Network Analysis

We visualized the social networks and analyzed them using different network metrics. The network size represents the number of individuals in the network without including the focal participant (*ego*). Strong ties are represented by red lines, denoting contacts who are more familiar with the participant and provide support *most often*, whereas weak ties are represented by blue lines (all other individuals). The mean degree is the average number of connections (ties) incident upon a member of the network. Effective size is the number of the focal node’s nonredundant neighbors, which is a function of the number of the focal node’s neighbors (*alters*) and the extent to which these neighbors are not directly connected to each other. The effective size varies from 1 (a network that provides only a single nonredundant contact) to the total number of ego neighbors (ie, each contact is nonredundant) [45]. Effective size represents the total value ego can extract from all its alters: the higher the effective size, the larger the number of nonredundant contacts, and the higher the benefit for the ego. The node representing ego in each network was associated with node strength, calculated as the sum of the weights of the ties connected to the node (no tie value=0, weak tie value=1, and strong tie value=2). The average tie weight was then calculated for each ego in the various networks by dividing the ego’s node strength by its degree. For each ego-centered network, the correlation coefficient between the ego’s average tie weight and the effective size was calculated using Microsoft Excel.

#### Qualitative Analysis

The workshops and interviews were audio-recorded, transcribed verbatim, and analyzed using thematic analysis. There were 2 parts to each transcript. The first covered the running of the social network assessment survey, and the second covered the participants’ answers to the following semistructured questions:

1. “How does your social network play a role in managing your health?”
2. “Do you ever read on internet what peers online say about living with your medical condition/s? Are you in contact with any peers online, e.g., on Facebook or the Asthma UK or BLF online communities?”
3. “If not, what would be the barriers and potential benefits?”

Transcripts were qualitatively analyzed using thematic analysis [48] to identify and define the emerging common themes. AA read all posts to familiarize with the data and the participants. ADS independently coded 20% of the data. Disagreements were identified between the coders. Following coding, the main themes and subthemes were identified, iteratively reviewed, and refined throughout the analysis. The results were triangulated with those from the quantitative analysis by comparing and combining them, contributing to one another. Emerging themes from participants’ comments during the social network assessment survey have been reported in the *Social Network Maps* section.

### Ethics Approval

The Queen Mary Ethics of Research Committee granted ethical approval before the start of the study (QMREC2388a). After the COVID-19 lockdown, an ethics amendment was sought to replace the second workshop with telephone interviews.

## Results

### Participant Characteristics

We recruited 7 participants aged between 64 and 81 years, of whom 5 (71%) were women, and 2 (29%) were men, with a mean age of 73 (SD 5.28) years. Of the 7 patients, 5 (71%) had COPD, and 3 (29%) had asthma (one of them had comorbid asthma and COPD). The baseline characteristics, including gender, age, and comorbidities, are presented in Table 1.

**Table 1:** Participants’ baseline characteristics.

Participant number	Gender	Age (years)	Married	Live alone	Condition
N1	Male	70-75	Yes	No	COPD^a^ and hypertension
N2	Female	60-65	No	Yes	COPD, asthma, and tachy-brady syndrome
N3	Female	75-80	Yes	Yes	Asthma, thyroid disease, and obstructive sleep apnea
N4	Female	65-70	Yes	No	Asthma
N5	Male	65-70	Not stated	Yes	COPD and ulcerative colitis
N6	Female	80-85	No	Yes	COPD and heart failure
N7	Female	75-80	No	Yes	COPD, interstitial lung disease, and hypothyroidism

^a^COPD: chronic obstructive pulmonary disease.

### Social Network Maps

**Figure 1:**
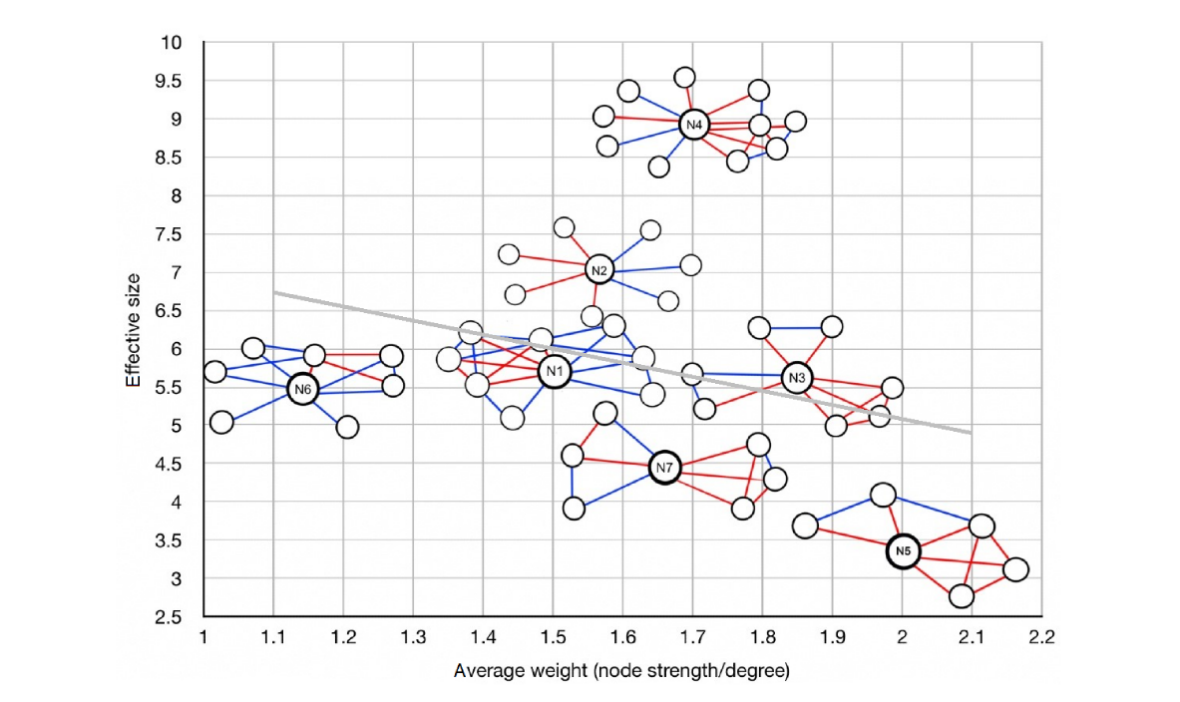
Social networks of participants arranged according to the ego’s effective size (y-axis) and average tie weight (x-axis). Line color indicates tie strength: red lines refer to strong ties and blue lines to weak ties. The gray line refers to the fitted regression line illustrating the relationship between effective size and average tie weight.

The use of the tool was feasible within the workshop and interview times and settings and acceptable to the 7 participants’ networks. Participants created network maps comprising between 5 and 10 individuals and individual groups, as shown in Figure 1, with network characteristics reported in Table 2 (a complete version of Table 2 is reported in the Multimedia Appendix 1). Of the 7 participants, 5 (71%) included close family members, considering them a major source of emotional support (strong ties, indicated by red lines in Figure 1). Family members can be recognized in the maps as mutually connected with strong ties. Participants who had close family members in London also reported that they were an important source of practical support, especially during exacerbations of their disease:

My son, well he lives in London and when I did have my exacerbation a couple of years ago, he went and did all my shopping and that sort of things because I couldn’t...

N3

Some participants belonged to multiple groups (eg, choirs, exercise groups, the singing groups) and found it easier to name the whole group than single individuals within them as playing a role in the management of their chronic diseases.

Participant N4 had the highest effective size (8.9) and thus a high number of structural holes (ie, the absence of direct links between contacts) in her network, which was, therefore, likely to provide access to novel nonredundant information more easily. Participant N5 had the most close-knit network with the lowest effective size (3.4) and the highest average tie weight, with most of his social contacts being connected to all others.

**Table 2:** Characteristics of social networks involved in the management of participants’ long-term respiratory condition.

Participant number	Network size^a^	Density^b^	Effect size^c^	Degree,^d^ mean (SD)	Maximum degree^e^	Kin proportion^f^	Average tie weight^g^	Named health care professionals	Any contacts on the web?
N1	8	39	5.7	2.7 (5)	5	37	1.50	GP^h^ and COPD^i^ nurse	No
N2	7	0	7	0^j^	0	14	1.57	Out-of-hours GP	Yes
N3	7	23	5.73	1.4 (2)	2	42	1.85	GP and practice chest nurse	No
N4	10	13	8.9	1.2	4	20	1.7	GP and nurses, pharmacists, and consultant	Yes
N5	5	50	3.4	2	3	60	2	None	Yes
N6	7	23	5.5	1.4 (2)	4	42	1.14	GP and respiratory care staff	No
N7	6	33	4.45	1.6 (6)	2	0	1.66	GP	No

^a^Total number of unique social contacts.

^b^Ratio of the number of ties to maximum possible number of ties.

^c^Effective size is the number of the ego’s nonredundant contacts based on the Burt measure.

^d^Average degree of a network member excluding the ego.

^e^Maximum degree of network member (most popular) excluding the ego.

^f^Proportion of network members who are kin.

^g^Node strength or degree.

^h^GP: general practitioner.

^i^COPD: chronic obstructive pulmonary disease.

^j^For some participants the tool could not calculate the SD and therefore only the mean is reported.

All participants had at least one friend in their social network who identified as a strong tie on the map. However, at least 50% of the members of the social networks of participants N2, N3, N4, and N7 were friends. These participants mentioned that in addition to emotional support, their friends offered them other forms of support, including life advice, practical and health tips, and financial support. Approximately 57% (4/7) of participants (N2, N3, N4, and N5) named their peers (ie, people with a chronic respiratory condition) among the network members. They stated that they felt part of a community when talking to peers about their disease and sharing information on how they were coping:

...the first was very much also finding out information, reading up, recommending exercises, so that’s one person...[with another friend] it’s good to have conversations where you’re talking about how you get online, you do things and how you are limited by whatever your condition is...

N4

The networks were plotted in terms of the ego’s effective size and average tie weight. The lower the ego’s effective size and the higher the average tie weight, the more close-knit and restricted the network. The correlation coefficient between effective size and average tie weight was −0.21. The negative correlation is consistent with the Granovetter [27] theory, according to which stronger triplets (ie, high average tie weight) tend to close up into triangles (ie, low effective size).

Of the 7 participants, 6 (86%) included health care professionals in their maps. Of these, only 14% (1/7) of them mentioned a respiratory consultant, whereas the rest mentioned their general practitioners (6/7, 86%) and respiratory nurses (4/7, 57%). These participants felt particularly close to their community clinicians and could rely on them for the management of their conditions, particularly during exacerbations or infections. In addition, most participants’ first line of contact with medical advice was with their community clinicians:

[The GP] is very supportive. I’m not on her doorstep every day, but she’s a very genuine person. She’s not what I call plastic. She’s very genuine. She’s very, very genuine and very caring...I would say [my GP provides] emotional, physical, health support

N7

The social maps of participants N2, N4, and N5 included contacts on the web. Only participant N2 was active on web-based social platforms before the lockdown restrictions because of the COVID-19 pandemic. N4 and N5 transitioned their offline contacts to the web because of lockdown restrictions. Participant N2’s online peers, in particular, provided emotional support (eg, during hospital admission for asthma exacerbation).

### Themes

**Figure 2:**
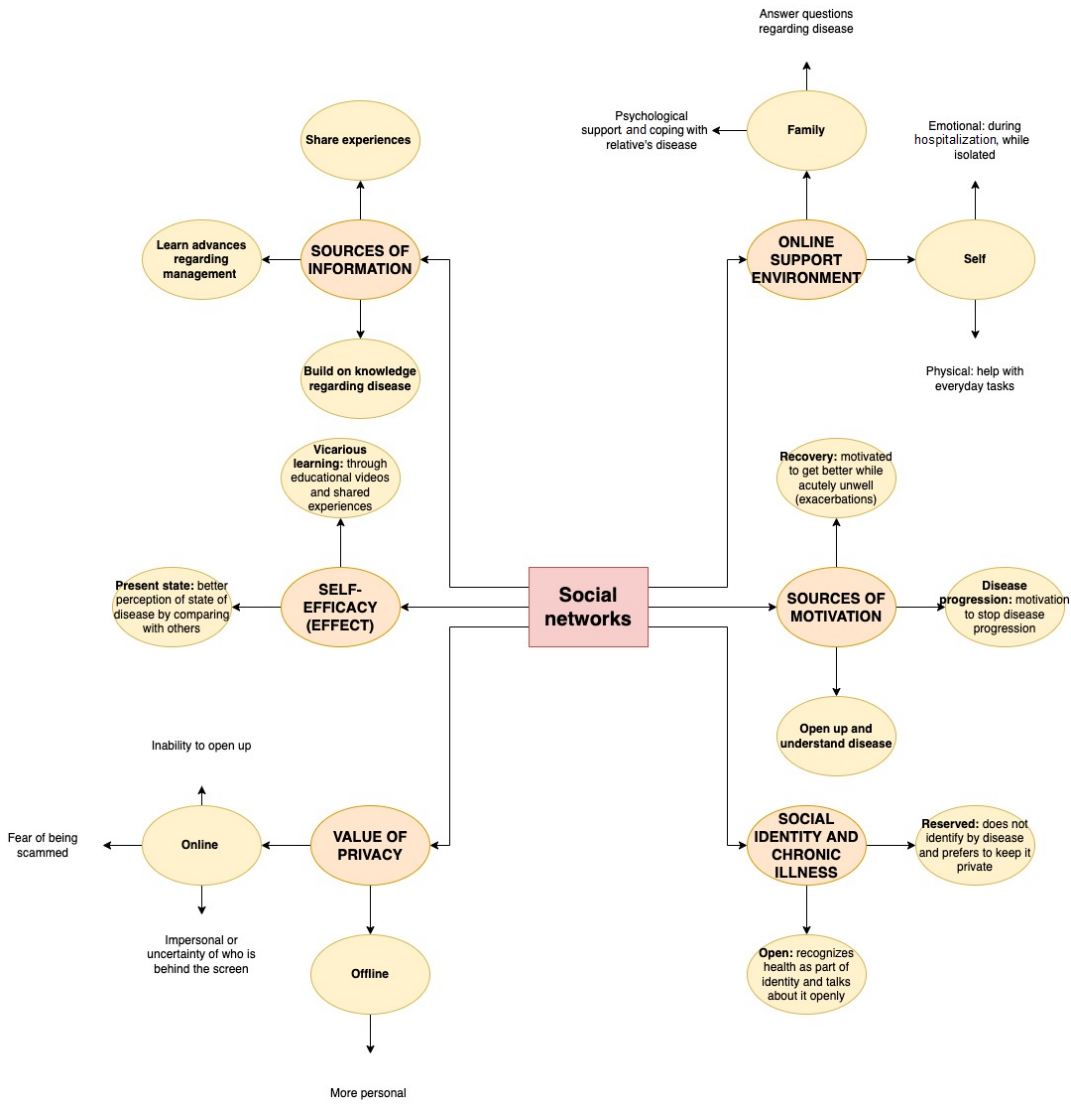
Main themes emerging from participants’ reflections on their social networks.

Several themes emerged regarding the role of both online and offline social networks in the management of chronic respiratory conditions, as illustrated in Figure 2.

#### Family and Chronic Illness

Most participants (5/7, 71%) included some close family members in their social networks. They highlighted the importance of family as a source of emotional support, as these are the people who know and understand them better, especially during exacerbations or when they face difficulties in their illness management. During the COVID-19 lockdown, participants reported that their close family members were still able to remotely provide the necessary support.

#### Social Networks and Self-efficacy

Wider social networks seemed to be markers of self-efficacy. Participants were able to better understand and manage their disease by accessing a broad social network that introduced them to novel information about self-management:

She’s a life coach and a, does energy healing and reiki and that, so that’s fantastic having her support, working on breathing and relaxation and that sort of thing...And another person is someone who I knew through work...they’re great because they’ve also got a network and they can recommend one, recommending a speech therapist or things like that

N4

#### Social Networks as Motivators and a Source of Perseverance

A major theme was concerned with social networks acting as motivators for improving one’s health. Participants were encouraged to open up and discuss their disease, improving their understanding and knowledge. They could make downward comparisons and monitor their disease trajectories with those of their peers, prompting healthier lifestyle choices that slow disease progression. In addition, participants noted that a strong and supportive social network encouraged them to recover swiftly during periods of being acutely unwell (ie, during exacerbations) and kept them engaged within their community:

...Some have had it longer than others, some are in a worse way than others. Obviously, there’s different stages so, and you can get an idea if you’re at a different stage. And you don’t want to go any further so you try your best to follow anything which could help your condition.

N5

#### Chronic Conditions and Social Identity

Some participants stated that, if possible, they preferred not to share their chronic health condition in their social networks and kept it restricted to their closest family and friends and health care professionals. Different traits regarding participants’ perceptions of disease and identity seemed to emerge through their reflections. Some participants were open to sharing their experiences and chronic illness, whereas others were more reserved, considering their illness somehow part of a more private sphere they would not easily talk about with others. This was because they avoided, if possible, talking about their health with others or *not to bother others*. This might suggest that the illness was not part of their identity, that they did not want the illness to dominate their life, or that they tried to reduce the emotional burden that living with the illness had on themselves and members of their social network:

I’m not a very..., my health is my health and I know what’s wrong with me and I just want to get on with it...I just have to get on with my own life, basically.

N6

Other participants were more open about their health and shared their illness experiences with others, including their peers. This allowed them to accept the support available to them from their network members in relation to emotional and practical work:

Friends at the church in general, yes. They really are wonderful. I was very ill with my lungs a couple of years ago and I used to get texts, phone calls, hi, how are you, do you need any help? It’s the same with this lockdown.

N7

### Benefits of Engagement With Online Peer Support

**Figure 3:**
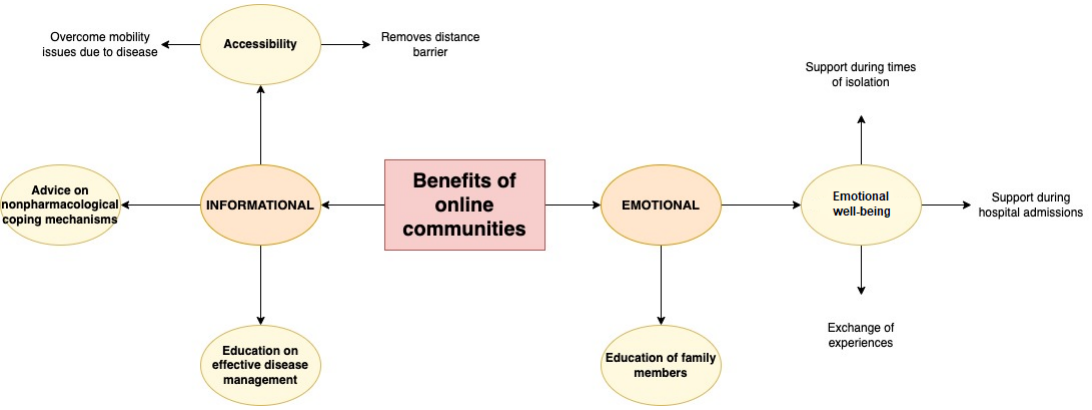
Benefits of online communities.

The benefits identified by participants of OHCs were grouped into informational and emotional support. These are illustrated in Figure 3.

Approximately 43% (3/7) of participants reported their experiences related to searching the web on breathing exercises, diet, meditation, and other calming techniques:

...people would be able to exchange more of those ideas and not feel embarrassed about saying, well actually, yeah, meditation really does help me to manage my breathing and to calm down and do that sort of, and lower anxiety levels, and if anxiety is a success, then it’s quite useful to get, keep your anxiety levels lower than.

N4

Participants described the potential of OHCs as a source of emotional support, fostering a sense of belonging and understanding. This benefit could be crucial when no other sources of support are available, such as during hospital admission, or when individuals are isolated or unable to attend face-to-face support groups and therefore are not able to benefit from sharing experiences or receiving the support of peers:

Immediately postop...I had the worst asthma attack I’ve had in years...But when I was in hospital, I was on Facebook then, I, all my friends, my friends were really supportive, I’d post updates and they came to see me...I was able to say, I’m getting better, or it’s going well or it’s not going well, so I got a lot of support.

N2

Online peer support can be readily accessible from the comfort of someone’s house, overcoming mobility constraints and eliminating the barriers of physical distance [49,50]. OHCs can be particularly useful at times of isolation, such as during the COVID-19 pandemic, and bring patients closer to their carers, peers, and family members:

...for whom making a journey was quite an effort, so it’s obviously, it’s the right sort of group for this group of people.

N4

Well with my group of friends, I couldn’t meet them because we’re located all over the country. With my family, yes I would only, I only use the Zoom because we can’t physically meet...

N6

Participants recognized the value of OHCs as informational or educational platforms in which people with chronic conditions can use peer support passively by either watching YouTube videos made by peers about self-management tips or reading what peers say. By accessing novel information, they could build on their knowledge regarding their disease management, undertake downward comparisons, and discover new methods of coping that would not otherwise be available:

Oh yeah, it’s definitely, I found it on YouTube, I don’t think I would get that information from any of the people in their [inaudible] as such.

N5

...I’ve actually seen videos of people who are in bad way with their oxygen and people like that who are trying to give you some, any information about how to cope if you get to that degree...I think to myself, I don’t want to end up like that, so I try and do everything I can possible until I’m [inaudible] getting worse.

N5

...I get too much mucus produced which sort of clogs me up a bit so I normally try and find out if I can, on YouTube, what sort of recipes they’ve got. And if I can, if they tell me, like ginger, garlic, rosemary, things like that which help with your breathing...

N5

Trust and practical everyday work around food and cooking were also reported:

Obviously, it’s quite a lot of good advice on there [online], in some areas it’s, some, you can take it with a pinch of salt...But most of it is pretty good information.

N5

### Barriers to Engagement With Online Peer Support

**Figure 4:**
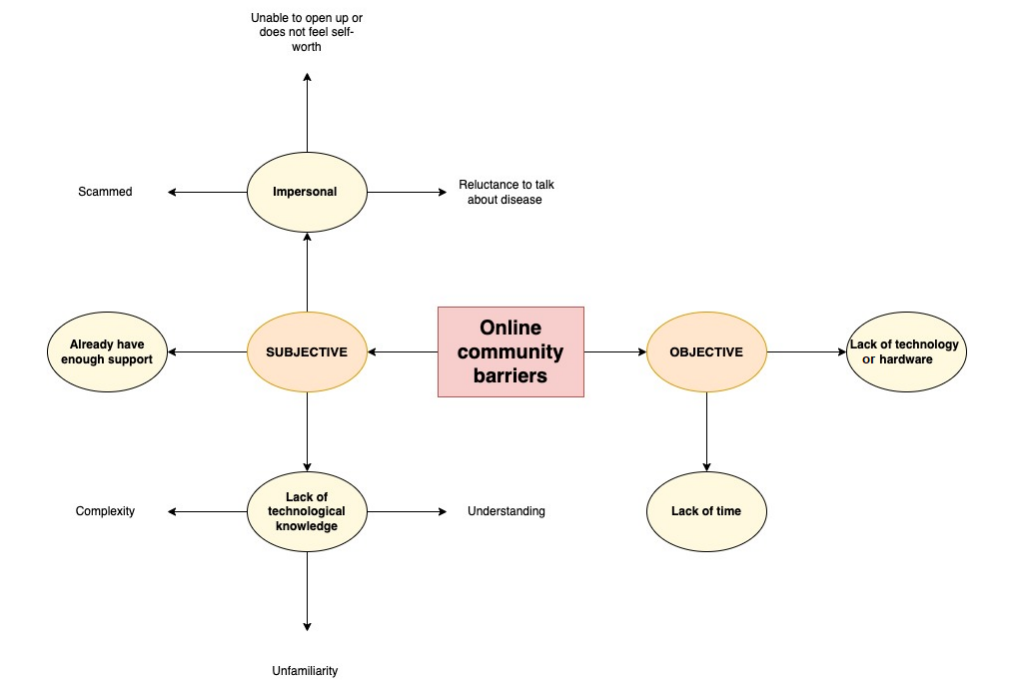
Barriers to online communities.

Barriers identified by the participants regarding engagement with OHCs in the context of illness self-management were grouped into subjective and objective. These are illustrated in Figure 4*.*

#### Subjective Barriers to Online Peer Support

Some participants felt worried that because of the remote nature of OHCs, relationships might not become as personal as during in-person interactions. Emerging themes included a lack of value added in relationships on the web, *interference of technology*, and a preference for not–web-based interactions. Participants might occasionally use the internet to search for information or clarify issues regarding their disease management, using it as quick and easy access to information not available via their existing network members. Their use of web-based resources could be seen as integrating access to existing offline relationships:

I don’t like, I don’t want to sit in front of my computer and read pages and pages and pages of stuff. I very, very rarely do that. I might look on Google for particular, something or other, and I might look at the information and think, good. But I’m not one to, I don’t watch...

N6

No, I never have. If there’s something I want to know I might Google to find out but other than that I will talk to people face to face if necessary.

N7

Similarly, a small number of participants were concerned about their ability to provide advice regarding health management because of lack of knowledge and expertise. This makes this group of participants even less likely to be involved in online peer groups:

No, I’m not prepared to help people as far as their health is concerned...there’s nothing that could compare to peoples’ health...It’s not my job. I don’t know enough about it to advise other people on what to do.

N6

One of the participants also expressed concerns about other risks on the web, not only as it is often difficult to understand people’s true motives behind a screen but also because of her lack of technological knowledge:

No, I’m not on Facebook. I’m not on Twitter. I’m not, I’ve never really been interested in all that actually. I’ve always been a bit scared I might get scammed in some way.

N7

The same participant explained that having an already well-established support network could be another limiting factor affecting engagement with OHCs:

But if I’ve got something wrong then I will book an appointment with the GP and I’m not on her doorstep every day fortunately enough. I’ve got too much respect for her, bless her.

N7

I’m quite happy with the support that I’ve got. If I had a problem, you see I mentioned I’ve got sleep apnoea as well. I have check-ups on that and Telly, the physio that I see sometimes...I can discuss it with her, and she’d give me advice on it because she’s so closely knit with all of them in respiratory medicine

N7

Some participants were cagey about the idea of making technology a feature of their lives, especially if they had been able to perform their everyday tasks without it previously. They mentioned how complicated technology could be for the older population or for anyone who had not used it regularly beforehand:

...if you have up till now been able to do something without the interference of technology and now you have to learn to do the same things that you’ve got to mediate with the technology, sometimes you think, oh I can’t be bothered with that...

N4

I actually had this conversation with someone before this morning, who was saying that when she... presses a button and things don’t happen as she expects them to do, she says, “oh, then I give up.” So, I think that people may have a certain level but can be quite easily discouraged and depending on how important it is...

N4

#### Objective Barriers to OHCs

Participants felt that not only did they lack the time required to familiarize themselves with the skills required to participate in OHCs, but they also did not have enough time to engage with them, even if they were able to navigate the web-based system:

My concern would be the about actually having the time to do it...

N4

Finally, some felt that the people who would truly benefit from OHCs are older patients who seem to experience more debilitating symptoms. However, these patients are least likely to be adequately equipped with the required technology, such as tablets, computers, and smartphones:

The only trouble with that is there’s a lot of people, like myself who have not really got any iPads or laptop, I’ve only had this one now for a year I think and I, it’s still pretty new to me...

N5

Participants highlighting barriers to engagement with OHCs tended to be those with low effective size, high tie weight, and a high percentage of network members being kin (ie, N7, N5, and N6), whereas benefits and openness to online peer support were indicated mainly by people with high effect size, low average tie weight, and low percentage of network members being kin (ie, N2 and N4).

## Discussion

### Principal Findings

This study shows the application of a web-based social network assessment tool in a population of patients with chronic respiratory conditions, extending it to include contacts both offline and on the web who were involved in their disease self-management. Participants were able to identify and visualize other people involved in the management of their chronic disease and describe the role of social networks in managing their health and illness, with the tool itself acting as a facilitator of this process, as previously shown [51]. With facilitation, using a web-based social network assessment tool was feasible and acceptable for this patient population in workshops and remote interview settings. The visualization of the social network through the tool enhanced participants’ understanding of the role of their engagement with online and offline social contacts in their disease self-management and the work undertaken by the network itself for people with asthma or COPD.

Our qualitative analysis showed that most respondents received emotional support from close family members and friends. A small minority of participants reported using peer support through OHCs, either when no other sources of support were available or passively through watching videos.

Participants with a higher effective size welcomed various forms of informational support and openly discussed their health with their friends and family. In addition, they belonged to different communities, with each group contributing informational support in a unique way and to a different extent. This suggests that the higher the effect size and the lower the node’s average tie weight, the more likely patients are to take advantage of the novel, nonredundant sources of information and support [28] and seek advice and help from others outside their close family network. This could directly affect, as well as improve, their illness self-management and self-efficacy [11,12]. These data highlight the value of using the social network assessment tool as an intervention that can support self-management when facilitated and as a research tool that can help assess and understand network structure and network engagement in the self-management support of people with chronic respiratory diseases.

### Interpretation in Light of Existing Evidence

Previous studies on social networks have shown that they can have broad effects on physical health outcomes and quality of life and even lower the risk of all-cause mortality [17,20-22]. There is also evidence to suggest that involvement in multiple support groups can foster an individual’s ability to self-manage and improve their well-being and ability to cope practically and emotionally [52]. In agreement with these reports, we found that the participants’ social contacts facilitated disease self-management and that networks were involved with different types of work (emotional and informational or behavioral), how the work was done, and by whom. In addition, our results are in line with the literature demonstrating a contagion effect of networks on behavioral change [23,24,26], as participants reported being encouraged by their social networks to take part in activities to keep them active and in better health conditions.

Several studies have explored the potential benefits of OHCs as health-related information sources [36,37] and how they can have a positive impact on health behavior–related outcomes [38]. It has been theorized that the extent of input by different members and OHCs of a network might change according to people’s current circumstances and relationships [6]. Participants welcomed the idea of OHCs as a source of information and readily accessible psychological aid, which removed the barriers of physical distance and isolation. However, some saw OHCs as impersonal, especially if they already had a strong offline network, and would not engage out of *fear of the unknown*. Similarly, some felt underequipped to engage in such communities because of their lack of technology and expertise.

### Clinical and Research Implications

The facilitated use of the social network tool [45] acted as an intervention that prompted reflections on offline and online social networks in the management of chronic respiratory conditions. This process of visualization and reflection has been referred to previously as a *positive disruption* [53]. Previous work has shown how network visualization and reflection lead to improved network engagement and can improve outcomes [54-56]. Future studies should investigate whether encouraging people with LTCs who are willing to engage in OHCs to expand their social networks to include online peers could enhance their access to novel information and potentially improve their self-management. In addition, given the direct impact that self-efficacy can have on self-management, OHCs could focus on connecting like-minded individuals and creating new relationships. Such interactions could also bolster conversations regarding health management and motivate individuals to achieve successful disease management. Our results showed that the COVID-19 pandemic created the need for social interactions on the web and equipped some participants with the skills to overcome technological barriers.

People’s perceptions and willingness to engage with OHCs are influenced by individual traits regarding willingness to discuss health and illness with others. Being able to identify patients’ preferences in terms of sharing illness self-management with peers could allow health care professionals to signpost them accordingly. Research programs informed by these activities can enhance patient-centered research on social media and health, with new significance in light of the social isolation caused by the COVID-19 pandemic. A longitudinal study of patient networks over time could help to understand how exogenous factors (such as pandemics) are associated with changes in people’s attitudes toward engagement with OHCs.

### Strengths and Limitations

The strengths of this study include testing the feasibility and acceptability of using a social network assessment tool [45] to visualize people involved in the management of chronic respiratory conditions in the United Kingdom. However, the small number of participants is a limitation that makes it difficult to extend and generalize the findings. Some participants named whole groups (eg, choirs and exercise groups) rather than single individuals as playing a role in the management of their chronic diseases, which may have introduced a bias in the quantitative assessment of the social network. Selection bias may have been introduced by recruiting participants who were already engaged in a health community through a singing group. Furthermore, the social maps were based on participants’ self-reported data; this raises the possibility of bias because of different cultural and personal beliefs on what individuals consider to be a *social network*. Finally, because of the COVID-19 pandemic and the more impersonal nature of the telephone interviews, participants’ responses might not reflect how they would have answered in a workshop.

### Conclusions

Online social networks are becoming increasingly important components of people’s everyday lives. Their appeal in the health care domain is not only attributable to their low cost but also to their potential for changing health behavior–related outcomes [38]. Our study opens new avenues for future research, including the investigation of the evolution of social networks overtime in people with chronic illnesses and, in particular, the association between the dynamics of engagement on the web in OHCs and illness self-management.

## Appendix

Multimedia Appendix 1 Complete data set (characteristics of social networks involved in the management of participants’ long-term respiratory condition).

## Abbreviations

COPD: chronic obstructive pulmonary disease
LTC: long-term condition
OHC: online health community
PPI: patient and public involvement
REDCap: Research Electronic Data Capture
