# Supplementary material for: Understanding Online and Offline Social Networks in Illness Management of Older Patients With Asthma and Chronic Obstructive Pulmonary Disease: Mixed Methods Study Using Quantitative Social Network Assessment and Qualitative Analysis
Source: JMIR Form Res. 2022 May 17;6(5):e35244. doi: 10.2196/35244 (PMC9157321; doi:10.2196/35244)
Supplement: Multimedia Appendix 1 [file formative_v6i5e35244_app1.docx]

| Participant number | Network size^a^ | Density^b^ | Effect size^c^ | Degree^d^, mean (SD) | Maximum degree^e^ | Kin proportion^f^ | Age (years)^g^, SD | IQV^h^ sex^i^ | IQV race^j^ | Weak frequency proportion^k^ | Weak duration proportion^l^ | Far distance proportion^m^ | Node strength^n^ | Average tie weight^o^ | Named health care professionals | Any contacts on the web? |
| --- | --- | --- | --- | --- | --- | --- | --- | --- | --- | --- | --- | --- | --- | --- | --- | --- |
| N1 | 8 | 39 | 5.7 | 2.75 | 5 | 37 | 12 | 0 | 46 | 25 | 50 | 75 | 12 | 1.50 | GP^p^ and COPD^q^ nurse | No |
| N2 | 7 | 0 | 7 | 0 | 0 | 14 | —^r^ | — | — | 28 | 71 | 28 | 11 | 1.57 | Out-of-hours GP | Yes |
| N3 | 7 | 23 | 5.73 | 1.42 | 2 | 42 | — | 88 | — | 29 | 14 | 42 | 13 | 1.85 | GP and practice chest nurse | No |
| N4 | 10 | 13 | 8.9 | 1.2 | 4 | 20 | 13 | 64 | 0 | 50 | 30 | 50 | 17 | 1.7 | GP and nurses, pharmacists, and consultant | Yes |
| N5 | 5 | 50 | 3.4 | 2 | 3 | 60 | 18 | 96 | 0 | 0 | 0 | 80 | 10 | 2 | None | Yes |
| N6 | 7 | 23 | 5.5 | 1.42 | 4 | 42 | 13 | 0 | 0 | 29 | 29 | 86 | 8 | 1.14 | GP and respiratory care staff | No |
| N7 | 6 | 33 | 4.45 | 1.66 | 2 | 0 | 6 | 96 | 0 | 60 | 80 | 100 | 10 | 1.66 | GP | No |

Table 2. Characteristics of social networks involved in the management of participants’ long-term respiratory condition – complete version.

^a^Total number of unique social contacts.

^b^Ratio of the number of ties to maximum possible number of ties.

^c^Effective size is the number of the ego’s nonredundant contacts based on the Burt measure.

^d^Average degree of a network member excluding the ego.

^e^Maximum degree of network member (most popular) excluding the ego.

^f^Proportion of network members who are kin.

^g^SD of network members’ age.

^h^IQV: index of qualitative variation.

^i^IQV of sex; higher number means balance of men and women in network.

^j^IQV of race; higher number means balance of race in network.

^k^Proportion of network members who contact ego: monthly or less often.

^l^Proportion of network members who have known ego for <6 years.

^m^Proportion of network members who live >15 miles away.

^n^Sum of the weights of links to/from a node (no tie value=0, weak tie value=1, and strong tie value=2).

^o^Node strength/degree.

^p^GP: general practitioner.

^q^COPD: chronic obstructive pulmonary disease.

^r^Data not available.
